# NLRC5 promotes tumorigenesis by regulating the PI3K/AKT signaling pathway in cervical cancer

**DOI:** 10.1038/s41598-024-66153-3

**Published:** 2024-07-04

**Authors:** Lin Ling, Jiahua Chen, Lei Zhan, Juanjuan Fu, Runhua He, Wenyan Wang, Bing Wei, Xiaofeng Ma, Yunxia Cao

**Affiliations:** 1 Department of Obstetrics and Gynecology, The First Affiliated Hospital of Anhui Medical University, Hefei, Anhui Province China; 2 Department of Obstetrics and Gynecology, The Second Affiliated Hospital of Anhui Medical University, Hefei, Anhui Province China

**Keywords:** NLRC5, PI3K/AKT, Cervical cancer, Prognosis, Autophagy, Cancer, Gynaecological cancer, Cervical cancer

## Abstract

Cervical cancer (CC) is the fourth most common cancer among women worldwide. NLR Family CARD Domain Containing 5 (NLRC5) plays an important role in tumorigenesis. However, its effect and mechanism in CC remains unclear. In this study, we aimed to investigate the function of NLRC5 in CC. NLRC5 was found to be down-regulated in CC tissues compared with normal cervical tissues. However, patients with higher NLRC5 expression had better prognosis, patients with higher age, HPV infection, lymph node metastasis, recurrence and histological grade had worse prognosis. Univariate and multivariate analyses showed NLRC5 to be a potential prognostic indicator for CC. Pearson correlation analysis showed that NLRC5 might exert its function in CC through autophagy related proteins, especially LC3. In vitro experiments demonstrated that NLRC5 inhibited LC3 levels and promoted the proliferation, migration, and invasion of CC cells by activating the PI3K/AKT signaling pathway. Treatment with LY294002 reversed the above phenotype. Taken together, our finding suggested that NLRC5 would participate in cervical tumorigenesis and progression by regulating PI3K/AKT signaling pathway. In addition, NLRC5 and LC3 combined as possible predictors in CC.

## Introduction

Cervical cancer (CC), one of the most common malignant tumors in the female reproductive system, has the fourth highest incidence rate in the world, causing 250,000 female deaths annually^1^. Although the morbidity and mortality rates of CC have decreased significantly due to major advances in the prevention, screening, and diagnosis of CC, the 5-year survival rate of patients with advanced CC after treatment is still low^2^. Therefore, it is urgent to explore the molecular mechanisms underlying the pathogenesis of CC to develop new strategies for the diagnosis and treatment of CC, especially for patients with advanced and recurrent CC.

NLRC5 is a newly identified member of the NLR family that acts as a transcriptional activator of MHC class I genes^3^. Recent studies have indicated that NLRC5 contributes to tumorigenesis by mechanisms beyond the regulation of MHC class I genes^4,5^. Previous studies have shown that NLRC5 promotes the proliferation, migration, and invasion in hepatocellular carcinoma, clear cell renal cell carcinoma, and endometrial cancer^6–9^. Mechanistically, a plethora of tumor related signaling pathways are involved in the regulation of NLRC5 in cancer. For example, He et al. showed that the over-expression of NLRC5 promoted cell proliferation by activating the PI3K/AKT/ vascular endothelial growth factor-A (VEGF-A) signaling pathway in hepatocellular carcinoma in vitro^7^. Up-regulation of NLRC5 has been shown to contribute to cell proliferation, migration and invasion in hepatocellular carcinoma and clear cell renal cell carcinoma by activating the Wnt/β-catenin signaling pathway in vitro and in vivo^6,8^. Recently, in an in vitro study in endometrial cancer, Fan and co-workers showed that the upregulation of NLRC5 expression induced cell migration and invasion by activating the PI3K/AKT signaling pathway^9^. Notably, the knockdown of NLRC5 in the renal proximal tubular epithelial cells significantly enhanced the activation of PIK3/AKT signaling pathway in hypoxia/reoxygenation-stimulated renal proximal tubular epithelial cells^10^. These findings demonstrated that the role of NLRC5 in the PIK3/AKT signaling pathway was cell-dependent. Nevertheless, the role and mechanism of NLRC5 in CC have not been explored.

Autophagy is an evolutionarily conserved mechanism, including the removal of damaged cellular components, which helps with the recycling and degradation of cytoplasmic components. Besides, it also enables the elimination of toxic factors and restores cellular homeostasis^11,12^. Thus, exploring the role of autophagy in tumor cells is of great significance for improving the effect of cancer therapeutics^13,14^. Researchers have identified a correlation between autophagy and NLRC5 expression. For example, in endometriosis, the overexpression of NLRC5 promotes the expression of LC3, Beclin1 and the formation of autophagosomes in the lesion, which inhibit disease progression^6^. PIK3/AKT signaling pathway is believed to be the the most important pathways that control autophagy^15^.

Collectively, we hypothesized that in patients with CC, NLRC5 might promote tumorigenesis by regulating PI3K/AKT signaling pathway. NLRC5 and autophagy combined as possible predictors in patients with CC. In this study, tissue microarray technique was used to investigate the expression of NLRC5, LC3 and Beclin 1 in CC tissues, to evaluate the association between the expression of NLRC5 and the clinicopathological parameters of the patients and to assess the correlation between NLRC5 expression and the prognosis of CC patients. Next, we evaluated the underlying mechanism associated with NLRC5 mediated effects in the progression of CC in vitro. Our study help increase the potential of approaches that regulating NLRC5 or autophagy level for use in therapy against CC.

## Materials and methods

### Tissue samples

CC tissue chip (HUteS154Su01) was purchased from Shanghai Xinchao Biotechnology Co., Ltd. The samples were from 119 patients with CC aged between 29 and 70 years old, of which 35 cases had matched corresponding peri-cancerous tissues. In addition, the detailed clinical information of patients, such as lifetime, tumor location, histologic differentiation, and tumor node metastasis (TNM) stage were also collected. According to the staging standard of primary cancer, lymph node and distant metastasis (TNM staging) were specified by the International Anti-Cancer Association (UICC), the degree of invasion was confined to the uterus (T1 stage) in 67 cases, and outside the uterus (T2-T4 stage) in 52 cases. 22 cases had lymph node metastasis and 97 cases had no lymph node metastasis. There was no distant metastasis in any of the tissue specimens. None of the patients underwent radiotherapy and chemotherapy before surgery. All treatments were approved by the Institutional Review Board of Anhui Medical University in this study. We confirmed that informed consent was obtained from all subjects and all research was performed in accordance with the Declaration of Helsinki.

### Immunohistochemistry

Tissue sections were routinely deparaffinized with xylene and rehydrated in decreasing concentration of ethanol. Antigen retrieval was achieved by boiling the sections in 10 mM citrate buffer for 20 min. After washing three time with PBS for 5 min each time, the sections were blocked with 3% hydrogen peroxide for 10 min to inhibit endogenous catalase. Then the sections were incubated with primary anti-NLRC5 antibody (ab105411, Abcam, diluted 1:100), anti-LC3 (ab192890, Abcam, diluted 1:100) and anti-Beclin 1 (ab210498, Abcam, diluted 1:100) overnight at 4°C. After washing with PBS, the sections were incubated with the horseradish peroxidase labelled secondary antibody at room temperature for 20 min. The sections were immersed in DAB (Beyotime, P0202) until they become colored and were then rinsed with running water. Finally, the sections were subsequently subjected to staining with hematoxylin, dehydration, permeation, resin sealing and were then photographed under the microscope.

### Evaluation of protein expression by immunohistochemical staining

**Figure 1:**
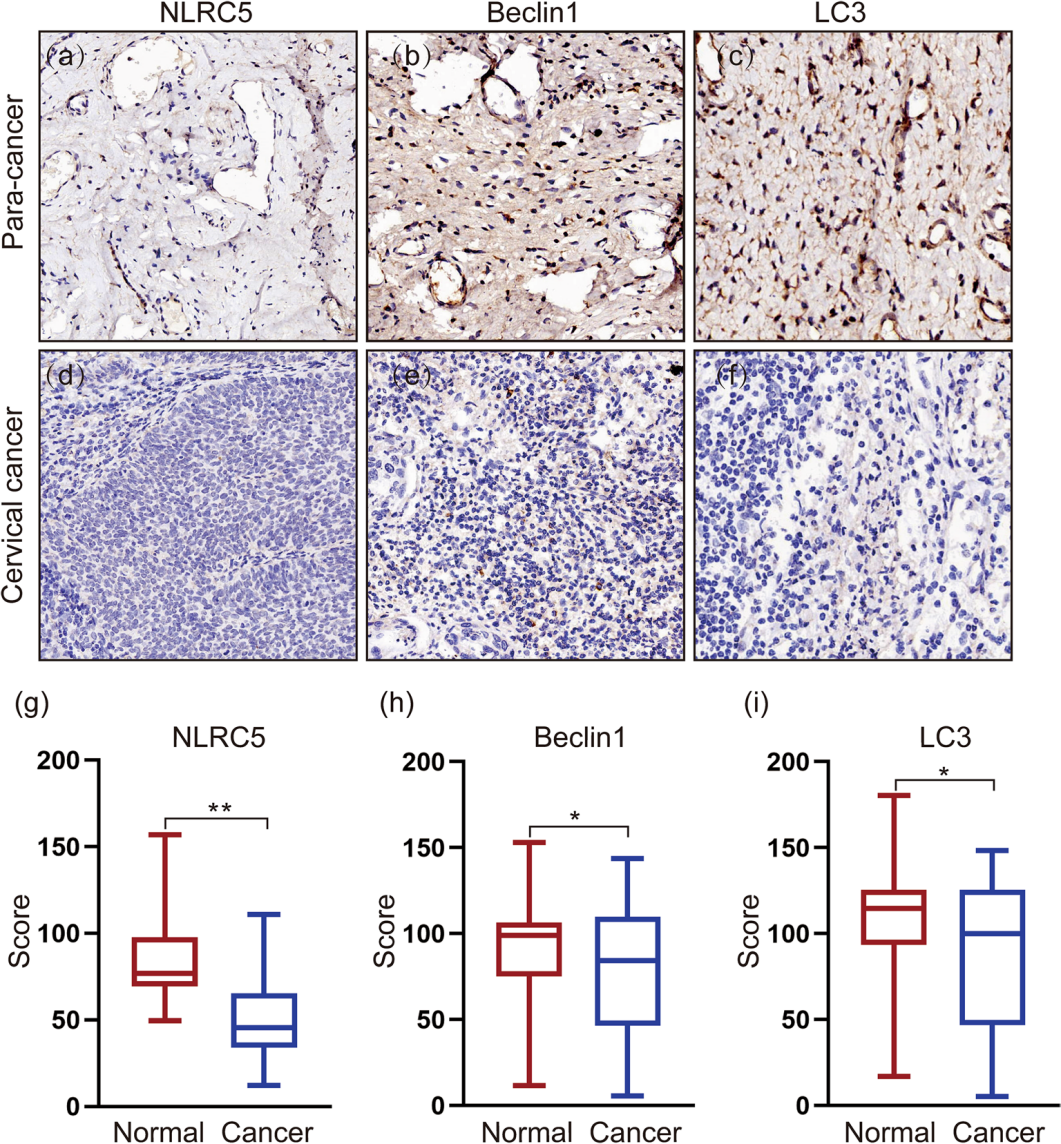
Immunoreactivity in cervical cancer and para-cancerous tissues. (magnification × 200). (**a**–**c**) IHC staining of para-cancer (n = 34). (**d–f**) IHC staining of cancer (n = 34). (**g**–**i**) Box plot of NLRC5, Beclin 1 and LC3 expression in cervical cancer (blue) and para-cancerous tissues (red). *p < 0.05, **p < 0.01, ***p < *0.001.*

IHC staining was evaluated by the histochemistry H Score on the basis of the extent and intensity of immunolabeling of the tumor sections. Briefly, the Seville image analysis system was used to automatically read the tissue measurement area, and the result was semi-quantitatively analyzed with the digital scoring system (H-score). H-Score = ∑(pi × i), where pi was the percentage of positive area and i was the density of positive signal. The density of staining was scored as 0 (absent), 1 (weak), 2 (moderate) and 3 (strong). The staining percentage of positive area was recorded from 0 to 100%. The H-score pattern of NLRC5 is shown in Fig. 1. The median of H-scores was the cutoff value. Final H-score > cutoff value was interpreted as high expression of NLRC5, while H-score ≤ cutoff value was interpreted as low expression of NLRC5^16^. The corresponding origin data in Supplementary Information 1.

### Cell culture and transfection

The human CC cell lines HeLa and SiHa were obtained from American Type Culture Collection (ATCC). CC cells were cultured in Dulbecco’s modified Eagle’s medium (DMEM, Invitrogen, Carlsbad, CA, USA) supplemented with 10% fetal bovine serum (HyClone, Logan, UT, USA), and 1% penicillin/streptomycin (Invitrogen) in the incubator at 37℃ under 5% CO2. For transfection with NLRC5 plasmid and small interfering RNA (siRNA)-NLRC5, the cells were treated with Lipofectamine 2000 (Invitrogen, USA) according to the manufacturer’s instructions. The full-length coding region of NLRC5 was created by using the primers F: 5′-CCGGAATTCCGGATGGCCAGGAAGCTGGA-3′, R: 5′-GGGATCCCGTCAC CTGAGTGTCTTC CCA-3′. siRNA-NLRC5 or scrambled sequences, which are listed in Table 1.

**Table 1:** The oligo sequences of siRNA-NLRC5 and scrambled for cell transfection.

Gene	Sequence (5′-3′)
NLRC5_F	AAGAACGAGAGACUCUG CCAACUGCdTdT,
NLRC5_R	GCAGUUGGCAGAGUCUCUCGUUCUUdTdT
Scrambled-F	UUCUCCGAACGUGUCACGUTT
Scrambled-R	ACGUGACACGUUCGGAGAATT

F, forward primer; R, reverse primer.

### Western blotting

Lysates were configured according to the ratio of RIPA (P0013K, Beyotime, China): phenylmethylsulfonyl fluorid (PMSF) (ST507, Beyotime, China):phosphatase inhibitor cocktail A(P1081, Beyotime, China) = 100:1:2 and CC cells were lysed with it. Protein concentration was measured using the bicinchoninic acid (BCA) kit (Sigma, USA). Lysates were collected by centrifugation at 12,000 rpm at 4 ℃ for 10 min. Equal amount of total protein was loaded on the different lanes of polyacrylamide SDS gels, after the proteins were separated, they were transferred onto PVDF membranes. The PVDF membrane was incubated with the primary antibody overnight at 4 ℃. The primary antibodies against NLRC5 (ab105411, Abcam, USA), p-AKT (Ser473) (AF0016, Affinity Biosciences), AKT (AF6261, Affinity Biosciences), PI3K (AF6241, Affinity Biosciences, Cincinnati, OH, USA), p-PI3K (Tyr607) (AF3241, Affinity Biosciences, Cincinnati, OH, USA), LC3 (ab192890, Abcam, USA) and GAPDH (GB12001, Servicebio, Wuhan, China) were used at 1: 1000, 1:1000, 1:1000, and 1:3000 dilution, respectively. Then the membranes were incubated in Tris-buffered saline-Tween (TBST; Boster Bio, Wuhan, China) containing 5% skim milk at room temperature for 1 h, followed by washing three times with TBS-Tween 20, and incubation with the horseradish peroxidase (HRP) labeled secondary antibody (Thermo Fisher Scientific, 1: 10,000) at room temperature for 1 h. Then, the membrane was washed thrice with TBST to remove the unbound secondary antibody. Electroluminescence (ECL, Thermo Fisher Scientific, Waltham, MA, USA) was used for detecting the signal from the protein bands. The images we provide all have corresponding images in the Supplementary Information 2.

### CCK-8 assay.

5 × 10^4^ cells/mL CC cells were plated on 12-well plates. Then 20 μL CCK-8 reagent was added into each well. The Synergy H4 Hybrid Microplate reader (Bio Tek, Winooski, VT) was used to measure the absorbance at 450 nm. The corresponding origin data in Supplementary Information 1.

### Cell migration and invasion assays

A 24-well Transwell Boyden chamber (Corning Inc., Corning, NY, USA) with an 8.0 μm pore size polycarbonate membrane was used for the migration and invasion assays according to the manufacturer’s instructions. In brief, CC cells were trypsinized and seeded in the upper chamber at a density of 1 × 10^5^ cells/well in 100 μL of serum-free medium. Then, 600 μL of complete medium was added to the lower chamber as a chemoattractant. After incubation for 24 h at 37 ℃, cells remaining at the upper surface of the membrane were removed with cotton swabs and the cells on the lower surface of the membrane were counted as the migrated cells. After fixation with 4% paraformaldehyde and staining with crystal violet solution, cells that passed through the filter were photographed by using an inverted fluorescence microscope. The invasion assay was performed in a similar manner, except that the transwells were pre-coated with 100 μL of 1:8 DMEM-diluted Matrigel (BD Biosciences, Franklin Lakes, NJ, USA) at 37 ℃ for 6 h before the cells were seeded onto the membrane, followed by incubation for 48 h.

### Treatment of CC cells with the PI3K broad-spectrum inhibitor LY294002

The PI3K broad-spectrum inhibitor LY294002 (Sigma Chemical Co., St. Louis, MO, USA) was dissolved in dimethyl sulfoxide (Sigma Chemical Co.). We used LY294002 at a concentration of 20 μM to inhibit the PI3K/AKT pathway in CC cells. The cells were first seeded overnight in culture dishes and transfected with NLRC5 plasmid 6 h later, followed by treatment with 20 μM LY294002 for 48 h.

### Statistical analysis

All data were analyzed using the SPSS 23.0 software (SPSS Inc., Chicago, USA). The difference in the expression levels of NLRC5, Beclin1and LC3 between the CC group and the corresponding normal tissue group were evaluated using the rank sum test. The median NLRC5, Beclin1 or LC3 expression was used as a cut-off value for grouping. Correlation analysis was performed using Pearson’s correlation. Kaplan–Meier survival curves were generated to investigate the prognostic significance of NLRC5, Beclin1 and LC3 in CC patients. Univariate and multivariate survival analysis were performed using log-rank and Cox risk regression model. All experimental data were expressed as mean ± SEM. *p* < 0.05 was statistically significant.

### Ethics approval

Ethical approval to report this data was obtained from the Institutional Review Board of Anhui Medical University (No.20180023). We confirmed that informed consent was obtained from all subjects and all research was performed in accordance with the Declaration of Helsinki.

## Results

### Expression of NLRC5 and autophagy-related proteins in cervical cancer

Human CC tissues (n = 34) and adjacent normal tissues (n = 34) were analyzed by immunohistochemistry to evaluate the expression levels of NLRC5, Beclin 1 and LC3 proteins. All cases presented staining for NLRC5, Beclin 1 and LC3 were predominantly detected in the cytoplasm of tumor cells. Apparently, normal adjacent to primary tumor presented the weak NLRC5, Beclin 1 and LC3 staining in cervical epithelium layers (Fig. 1a–f). Statistical analysis based on the H-score estimated in IHC staining revealed a significant difference in NLRC5 expression between the CC tissues and para-cancer tissues (z = 4.796, *p* < 0.01). Similar results were also observed for Beclin 1 (z = 20,129, *p* = 0.033) and LC3 (z = 2.207, *p* = 0.027) IHC staining cases (Fig. 1g–i).

### Correlation between the expression of NLRC5 and clinical characteristics of cervical cancer patients

To investigate whether NLRC5 expression was associated with the progression of CC, we analyzed the correlation between NLRC5 expression and the clinicopathological features (Table 2). The chi-square test revealed a significant association of NLRC5 expression with the histological subtypes, where tumors with lower NLRC5 expression were more often squamous carcinoma subtype (*p* = 0.044). No significant relationship was found between NLRC5 expression and the other listed clinicopathological parameters such as age (*p* = 0.168), histological grade (*p* = 0.168) and HPV infection (*p* = 0.168).

**Table 2:** Association between levels of NLRC5 with clinicopathological variables.

Variable	Patients (n)	NLRC5 expression		χ^2^	p
		Low N (%)	High N (%)		
Age (years)				1.898	0.168
≤ 45	58	33 (55%)	25 (42.3%)		
> 45	61	27 (45%)	34 (54.7%)		
TNM stage				0.203	0.652
TNM 1	67	34(54.7%)	33 (55%)		
TNM 2–4	52	25(42.3%)	27 (45%)		
Lymphatic metastasis				0.002	0.965
Absent	97	48 (81.4%)	49 (81.7%)		
Present	22	11 (18.6%)	11 (18.3%)		
Recurrence				0.422	0.516
Present	39	18 (30%)	21 (35.6%)		
Absent	80	42 (70%)	38 (64.4%)		
Histological subtype				8.114	0.044
Squamous carcinoma	110	53 (91.4%)	57 (95%)		
Adenocarcinoma	2	0 (0%)	2 (3.3%)		
Squamous adenocarcinoma	5	5 (8.6%)	0 (0%)		
Other	1	0 (0%)	1 (1.7%)		
HPV infection				0.236	0.888
High-risk type	57	30 (50.8%)	27 (45%)		
None high-risk	46	22 (35.6%)	24 (41.7%)		
Uninfected	16	8 (13.6%)	8 (13.3%)		

### Survival analysis reveals a positive correlation between NLRC5 expression and the prognosis of cervical cancer patients

**Figure 2:**
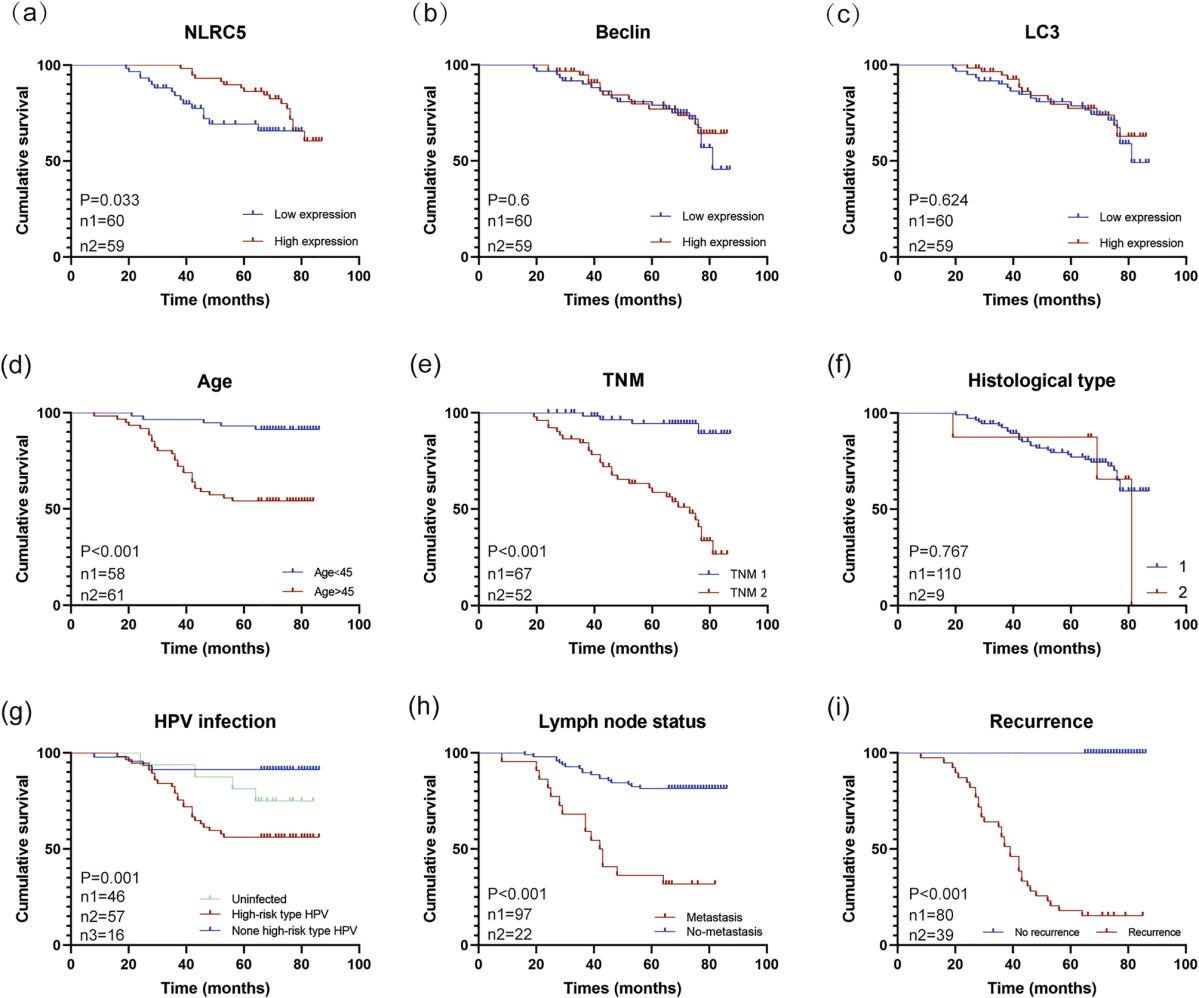
Kaplan–Meier survival curves of cervical cancer patients. (**a**) NLRC5 (χ^2^ = 4.55, *p* = 0.033), (**b**) Beclin1 ((χ^2^ = 0.274, *p* = 0.6), (**c**) LC3 (χ^2^ = 0.240, *p* = 0.624), (**d**) Age (χ^2^ = 18.853, *p* < 0.001), (**e**) TMN stage ((χ^2^ = 31.295, *p* < 0.001), 1 represents TNM 1, while 2 represents TNM 2–4. (**f**) Histological type (χ^2^ = 0.088, *p* = 0.767). 1 represents squamous carcinoma, while 2 represents adenocarcinoma, mixed squamous and adenocarcinoma and others. (**g**) HPV infection (χ^2^ = 14.949, *p* = 0.001), (**h**) Lymph node status (χ^2^ = 23.846, *p* < 0.001), (**i**) Recurrence (χ^2^ = 95.207, *p* < 0.001).

To evaluate the prognostic role of NLRC5 in CC patients, disease free survival (DFS) rate was determined by Kaplan–Meier survival analysis (Fig. 2). Log rank test demonstrated that patients with high NLRC5 expression had a significantly longer DFS than those with low NLRC5 expression (Fig. 2a). Among the clinicopathological characteristics, patients with young age, low TNM staging, no metastasis, no HPV infection and no recurrence had better outcomes (*p* < 0.05) (Fig. 2d, e, h, i). However, there was no statistically significant association between the expression of Beclin1 (*p* = 0.6), LC3 (*p* = 0.624), histological type (*p* = 0.767) and overall survival (OS) (Fig. 2b, c, f).

The Cox proportional hazard model was used for univariate and multivariate analyses to assess the effects of NLRC5, Beclin1, LC3 and clinicopathological variables in CC patients (Table 3). The univariate analysis showed that NLRC5 expression level, age, TNM stage, lymph node status and recurrence in CC patients were correlated with patient prognosis (*p* < 0.05). The variables with statistically significant differences in univariate analysis were included in the Cox multivariate survival regression analysis and the results showed that the level of NLRC5 expression, age and TNM stage were associated with patient prognosis (*p* < 0.05). In other words, patients with younger age, early stage and high NLRC5 expression had better prognosis. These results suggest that NLRC5 may potentially be one of independent prognostic factors in CC.

**Table 3:** Univariate and multivariate Cox regression analysis of clinicopathological parameters and prognosis in patients with cervical cancer.

Parameter	n	Univariate regression			Multivariate regression		
		HR	96% CI	p	HR	96% CI	p
Age (year)					9.156	3.35–25.05	0.000
≤ 45	58	6.26	2.42–16.23	0.000			
> 45	61						
TNM stage							
TNM 1	67	10.76	3.78–30.63	0.000	12.59	4.30–36.05	0.000
TNM 2–4	52						
T stage							
T1	67	10.76	3.78–30.63	0.000			
T2-4	52						
N stage							
N0	97	4.76	2.38–19.50	0.000			
N1	22						
M stage				–			
M1	119						
M2	0						
Lymph node status							
No	97	4.76	2.381–9.497	0.000			
Metastasis	22						
Recurrence							
Present	39	387.75	7.58–19,848.31	0.003			
Absent	80						
NLRC5 level					0.24	0.10–0.53	0.000
Low	60	0.46	0.22–0.96	0.037			
High	59						
LC3 level							
Low	60	0.84	0.42–1.68	0.626			
High	59						
Beclin level							
Low	60	0.83	0.42–1.66	0.602			
High	59						
Histological subtype							
Squamous arcinoma	110	1.20	0.36–3.92	0.769			
Others	8						
HPV							
Uninfected	16						
High-risk type	57	1.56	0.54–4.50	0.408			
High-risk	46	0.25	0.06–1.01	0.051			

### The correlation of NLRC5 expression, Beclin 1 expression and LC3 expression

Correlation analysis was performed to understand the potential relationship of NLRC5, LC3 and Beclin 1 expression (Table 4). We found no correlation between NLRC5 and Beclin 1 expression. However, NLRC5 expression was weakly and positively correlated with LC3 expression (R = 0.384, *p* < 0.001) indicating that NLRC5 may exert its function in CC through LC3. LC3 expression was also positively correlated with Beclin 1 expression (R = 0.434, *p* < 0.001).

**Table 4:** Relationship (Person R-value) of NLRC5 expression, Beclin 1 expression and LC3 expression.

Correlation (p-value)	NLRC5	Beclin1	LC3
NLRC5	1 (n.a.)	0.157 (0.089)	0.384 (< 0.001)
Beclin1	0.157 (0.089)	1 (n.a.)	0.434 (< 0.001)
LC3	0.384 (< 0.001)	0.434 (< 0.001)	1 (n.a.)

n.a., not applicable.

### Overexpression of NLRC5 induces proliferation, migration, and invasion of CC cells in vitro

**Figure 3:**
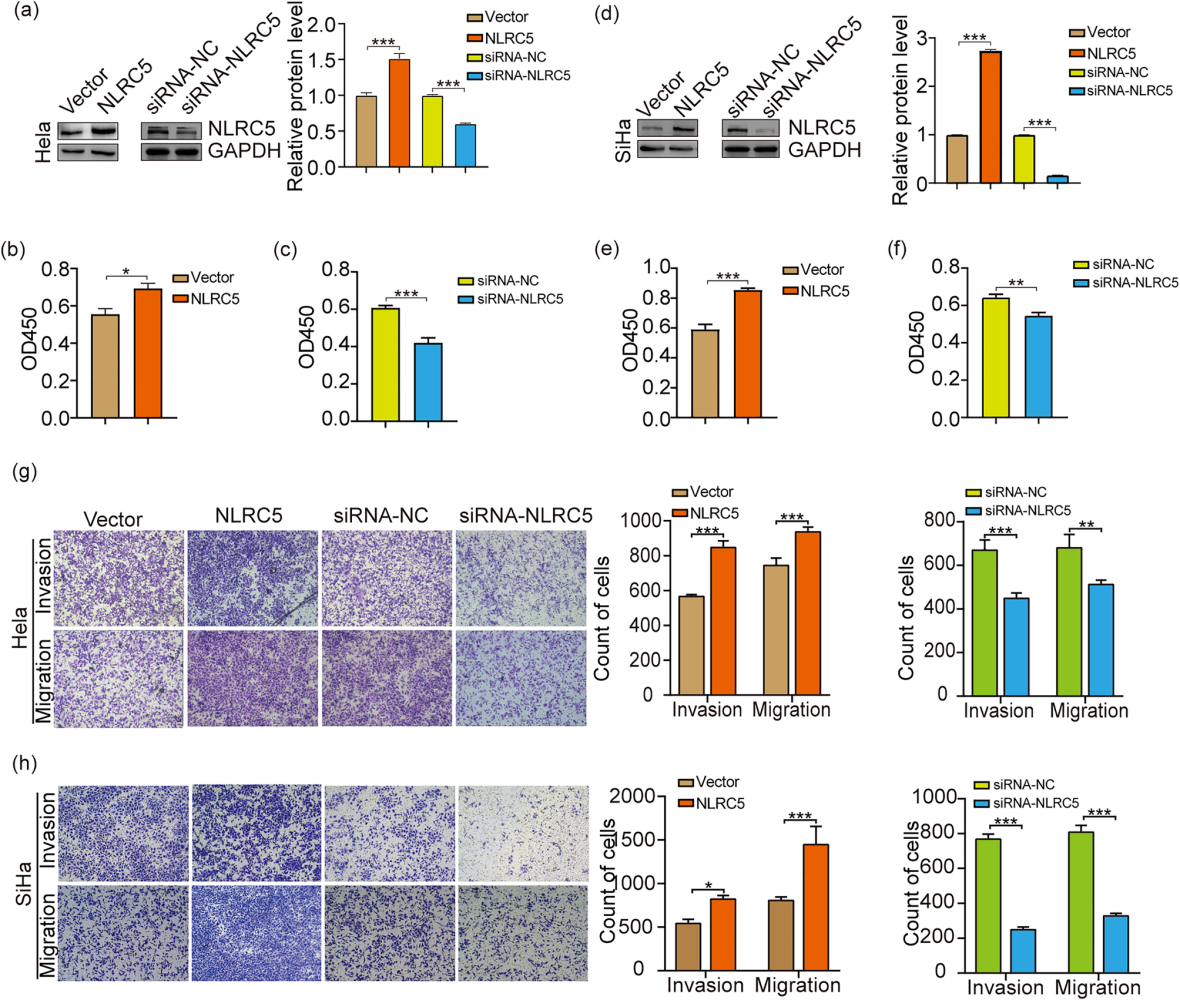
NLRC5 overexpression led to CC cell proliferation, migration, and invasion. (**a**, **d**) WB detected the protein expression in CC cell with NLRC5 overexpression or knockdown. (**b**, **c**, **e**, **f**) CCK8 assay detected cell proliferation after overexpression or knockdown of NLRC5. (**g**, **h**) Transwell assay detected cell invasion after overexpression or knockdown of NLRC5. *p < 0.05, **p < 0.01, ***p < 0.001. The results are represented as the mean ± SEM from at least three independent experiments.

To investigate the function of NLRC5 in CC, HeLa and SiHa cells were transfected with plasmid encoding NLRC5 or siRNA for NLRC5 (Fig. 3a, d). After transfection, CCK-8 (Fig. 3b, c, e, f), migration, and invasion assays (Fig. 3g, h) were conducted to assess cell viability, migration, and invasion. As shown in Fig. 3b, e, g and h, the over-expression of NLRC5 increased the proliferation, migration, and invasion of CC cells (*p* < 0.01). On the contrary, the above phenotype was inhibited upon the knockdown of NLRC5 in CC cells with siRNA (Fig. 3c, f, g and h, p < 0.01).

### NLRC5 regulates LC3 expression to promote CC cell proliferation, migration and invasion by activating the *PI3K/AKT Signaling* pathway

**Figure 4:**
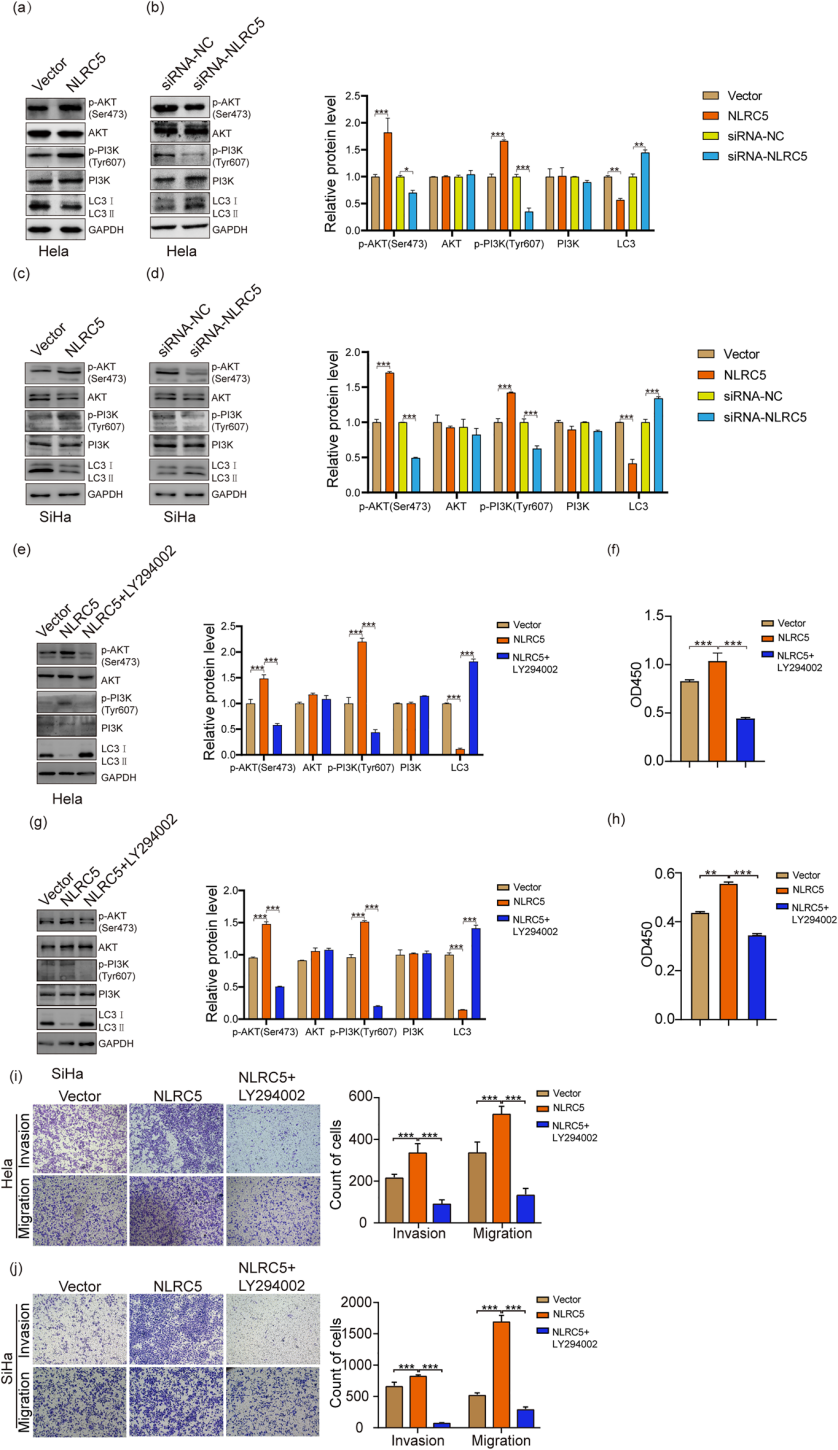
NLRC5 potentially regulates proliferation, migration, and invasion of CC cells by modulating LC3 expression and activating the PI3K/AKT signaling pathway. (**a**–**d**) WB showing the expression of PI3K/AKT pathway related proteins in CC cells with NLRC5 overexpression or knockdown. (**e**, **g**) WB showing the expression of PI3K/AKT pathway related proteins in CC cells with NLRC5 overexpression and LY294002 treatment. (**f**, **h**) CCK8 assay showing the viability of NLRC5 over-expressed CC cells after treated with LY294002. (**i**, **j**) Transwell assay showing the invasion potential of NLRC5 over-expressed CC cells after treated with LY294002. *p < 0.05, **p < 0.01, ***p < 0.001. The results are represented as the mean ± SEM from at least three independent experiments.

To explore the underlying molecular mechanism associated with NLRC5 mediated tumorigenesis in CC, we explored its effects on the PI3K/AKT signaling pathway by Western blotting. Over-expression of NLRC5 promoted the activation of the PI3K/AKT signaling pathway in CC cells (Fig. 4a, b, p < 0.01). Knockdown of NLRC5 expression decreased the expression of p-PI3K and p-AKT in CC cells (Fig. 4c, d). Moreover, after treatment with LY294002, an inhibitor the PI3K/AKT signaling pathway, the NLRC5 mediated effects on the proliferation, migration and invasion of CC cells was suppressed (Fig. 4f, h, i, j).

In consideration of there was a positive correlation between NLRC5 and LC3 expression. We further investigate the effects of NLRC5 on LC3 expression. We found over-expression of NLRC5 inhibited the expression of LC3 in CC cells (Fig. 4a, c). Knockdown of NLRC5 expression promoted the expression of LC3 in CC cells (Fig. 4b, d). Additionally, the negative effect of NLRC5 on LC3 expression were rescued after treatment with LY294002 (Fig. 4e, g).

## Discussion

The innate immune system is involved in the recognition and removal of invading pathogens by using germline encoded pattern-recognition receptors (PRRs), which can recognize intracellular pathogen-associated molecular patterns (PAMPs) and danger-associated molecular patterns (DAMPs)^17^. NLRs are a family of evolutionarily conserved cytoplasmic PRRs that mainly play key roles in immunity and host defense^18^. NLRs have a typical tripartite structure, including a centrally located nucleotide binding oligomerization domain (NBD) that is involved in oligomerization upon ligand recognition, the C-terminal leucine-rich repeats (LRRs) that can recognize the activators, and a variable N-terminal effector domain that mediates downstream signaling of activated NLRs^19^. NLR proteins are mainly localized in the cytoplasm, and have been shown to recognize pathogens and induce the production of multiple cytokines and chemokines^20^. Consequently, the conserved members of NLR family play major roles in the detection of invading pathogens and triggering immune responses toward bacterial and viral challenge. They do so by activating the NF-κB and MAPK signaling pathways, type I interferon signaling, and inflammasome signaling to activate procaspase-1 or secrete IL-1β^21,22^.

NLRC5 possesses the longest leucine-rich repeat domain protein among all human NLRs and regulates inflammation and immune response^21,22^. It has three structural domains, including the N-terminal atypical caspase activation and recruitment domain, which makes it completely different from the other NLRs, the central NACHT domain containing the nucleotide-binding domain (NBD) and the C-terminal containing Leucine repeats. Recently, accumulating evidence has shown that NLRC5 is intimately involved in the development of tumors. For example, NLRC5 is up-regulated in hepatocellular carcinoma and renal carcinoma and contributes to their tumorigenesis^6,8^. Interestingly, NLRC5 is down-regulated in endometrial cancer, however, the over-expression of NLRC5 promotes the migration and invasion of endometrial cancer cells^9^. Therefore, the level of NLRC5 expression in cancer is tissue-dependent. In the current study, the level of NLRC5 in human CC tissues was significantly lower than that in the normal cervical tissues. However, we found up-regulation of NLRC5 induced proliferation, migration and invasion of CC cells. Accordingly, the knockdown of NLRC5 expression suppressed the above phenotype. In humans, epigenetic changes like aberrant methylation at the CpG islands within the promoter regions of tumor associated genes represents one of the vital mechanisms to control tumor growth and immune escape^23^. Yoshihama et al. found that aberrant methylation in tumor cells resulted in the suppression of the mRNA levels of NLRC5^24^, which might explain the difference in the expression levels of NLRC5 in normal cells and CC tissues. In addition, the expression of NLRC5 is also influenced by the tumor immune microenvironment^24^.

Tumorigenesis is closely associated with the activation of pro-tumorigenic signaling pathways. Activation of the PI3K/AKT signaling pathway is known to be a classical event in the development of cancer, including CC^25,26^. Notably, the PI3K/AKT signaling pathway was reported to be involved in NLRC5 regulation, but the results were controversial. NLRC5 was reported to positively regulate the PI3K/AKT signaling pathway in endometrial cancer and hepatocellular carcinoma^7,9^, but it was the opposite case in acute kidney injury^10^. Until now, it is unclear how NLRC5 modulates the PI3K/AKT signaling pathway. Our study in the CC cells indicated that NLRC5 might activate the PI3K/AKT signaling pathway. Furthermore, NLRC5 accelerated CC cell proliferation, migration, and invasion potentially by activating the PI3K/AKT signaling pathway, while treatment with the PI3K/PI3K inhibitor LY294002, attenuated the NLRC5 induced effects of the proliferation, migration, and invasion of CC cells.

Autophagy is a highly regulated and major catabolic process in the cell where cytoplasmic substances are degraded within the lysosome by hydrolases and the resulting degraded products are recycled back to the cytoplasm to maintain cellular homeostasis^27^. LC3 and Beclin1 are essential autophagy-related gene (ATG) proteins, involved in different stages of autophagy^28^, and have been found to be directly associated with cancer. LC3 is the marker of autophagosome and consists of two forms, namely, LC3-I and LC3-II. LC3-II exists on the outer and inner membranes of autophagosomes and is positively associated with the level of autophagy in cells. Beclin1 contributes to the formation of the phosphatidylinositol-3-OH kinase-Beclin1-VPS34 complex during the initiation of autophagy^29^. The relation between autophagy and immunity is complex^30,31^. Autophagy has been reported to promote the internalization and degradation of MHC class I in dendritic cells, while autophagy-deficient dendritic cells enhance the presentation of different viral antigens to CD8+ T cells^32^. Meanwhile, immune signals regulate autophagy pathways in the tumor microenvironment, and these signals are perceived by recognition receptors inside or outside the cell membrane, including TOLL-like receptors, which are thought to be the inducers of autophagy and are involved in the activation of NF-KB dependent transcription to activate autophagy^33^.

Autophagy plays a unique role in cancer, which has been described as a “double-edged sword”^34,35^. Jan Karlseder and co-workers reported that autophagy suppressed tumor progression by restricting chromosomal instability^36^. However, Dou et al. suggested that autophagy might have an opposite effect because defects in autophagy could interfere with cellular senescence and limit the proliferation of damaged cells, leading to abnormal proliferation of cancer cells and ultimately promoting tumor progression^37^. The tumor promoting or inhibiting effects of autophagy depends on the tumor microenvironment^38^, which may explain the negative correlation between NLRC5 and LC3 expression levels in CC cells. Of note, NLRC5 expression has been closely associated with autophagy. For example, it was reported that there was a negative correlation between NLRC5 expression level and autophagy in endometriosis, suggesting the prognostic potential of the combination of NLRC5 and autophagy in patients with endometriosis^29^. A recent study by Zhan and co-workers illustrated that LC3 interacted with NLRC5 and further inhibited NLRC5-mediated MHC class I antigen presentation pathway in endometrial cancer^39^.In the current study, we found that the expression of Beclin1 and LC3 were down-regulated in CC tissue. Importantly, Pearson analysis showed that NLRC5 was positively correlated with LC3 expression level, which indicated that NLRC5 may be involved in CC by regulating LC3. Furthermore, we found NLRC5 inhibited the expression of LC3 through activating PI3K/AKT signaling pathway, indicating that NLRC5 and LC3 combined as possible predictors in patients with CC.

Collectively, we found that NLRC5 would participate in cervical tumorigenesis and progression by activating PI3K/AKT signaling pathway, so that NLRC5 can be a promising therapeutic target for the treatment of CC in the clinic. Furthermore, NLRC5 was positively correlated with LC3 expression level in CC, NLRC5 could inhibit the expression of LC3 through activating PI3K/AKT signaling pathway, indicating that NLRC5 and LC3 combined as possible predictors in patients with CC. However, the expression of NLCR5 is low in CC tissues, contradictory to that the overexpression of NLRC5 promotes cell proliferation and migration, which may be related to the fact that the components of CC tissues are very complex, and the immune cell components of CC tissues may affect the expression of NLRC5. Therefore, we speculate whether the contradictory results of this study are caused by the different microenvironments of tumor cells. Some studies have shown that certain specific mechanisms of NLRC5 depend on its functional regions, but whether the effects of these functional regions are related to the tumor microenvironment is also unclear. In order to solve this problem, we need to explore the role of NLRC5 in the immune microenvironment of CC, and to observe whether the NLRC5 functional region is selectively involved in different regulatory mechanisms. Besides, the number of samples we assessed was not enough large to permit analysis of other confounding variables. Lastly, the autophagy activity in our present study is unclear because of only LC3 expression was detected, investigation of autophagy flux could help clarify the role of autophagy in CC. Both of these points need to be further demonstrated in our subsequent experiments.

### Supplementary Information

Supplementary Figures.

Supplementary Information 2.

## Data Availability

The data that support the findings of this study are available on request from the corresponding author Xianfeng Ma upon reasonable request.
