# Supplementary material for: NLRC5 promotes tumorigenesis by regulating the PI3K/AKT signaling pathway in cervical cancer
Source: Sci Rep. 2024 Jul 4;14:15353. doi: 10.1038/s41598-024-66153-3 (PMC11222428; doi:10.1038/s41598-024-66153-3)
Supplement: Supplementary file 2 — Supplementary Information 2. [file 41598_2024_66153_MOESM2_ESM.pdf]

# Original uncropped images of western blots used for the experiment

Related to Figure 3a

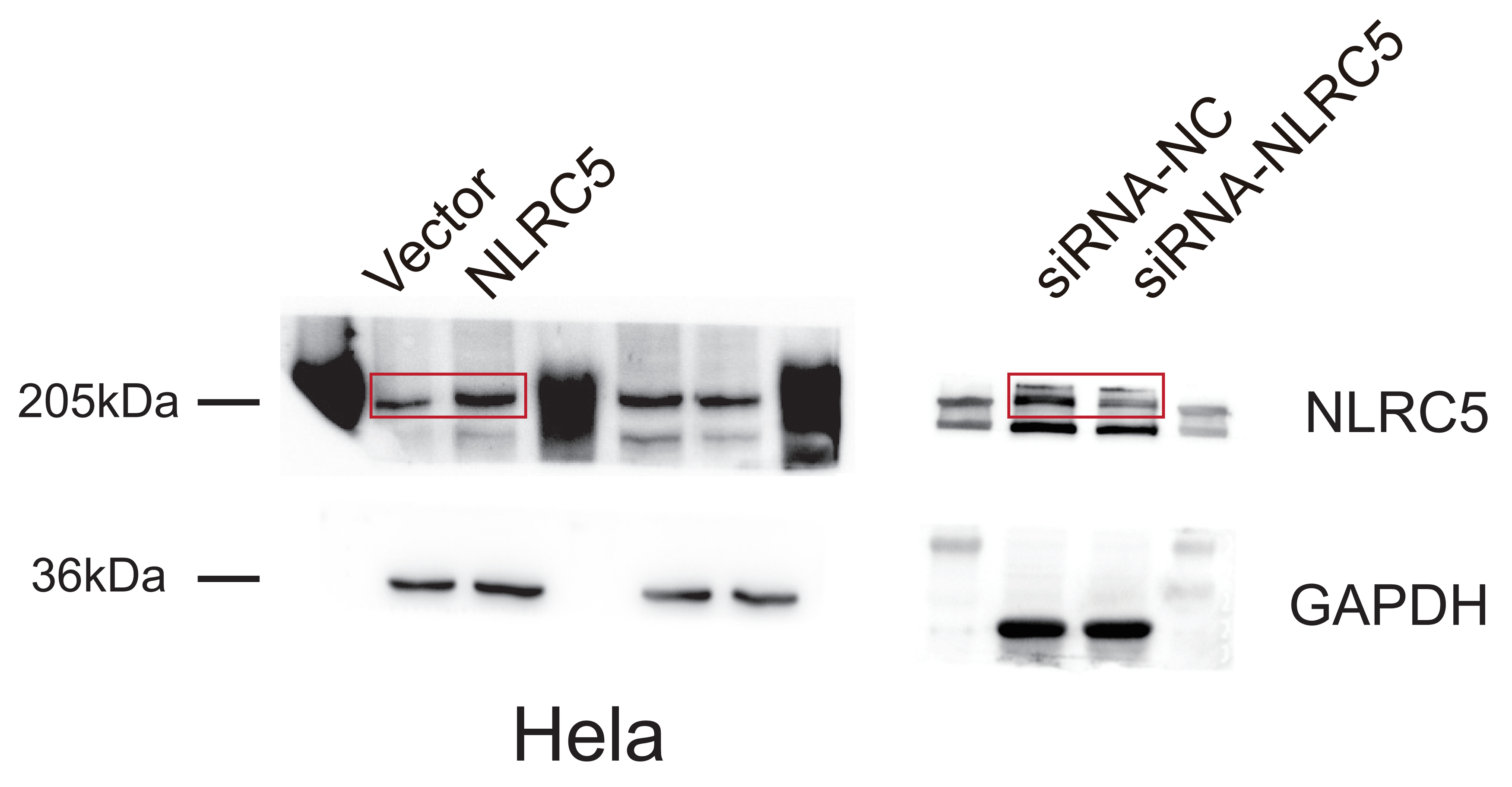


Related to Figure 3d

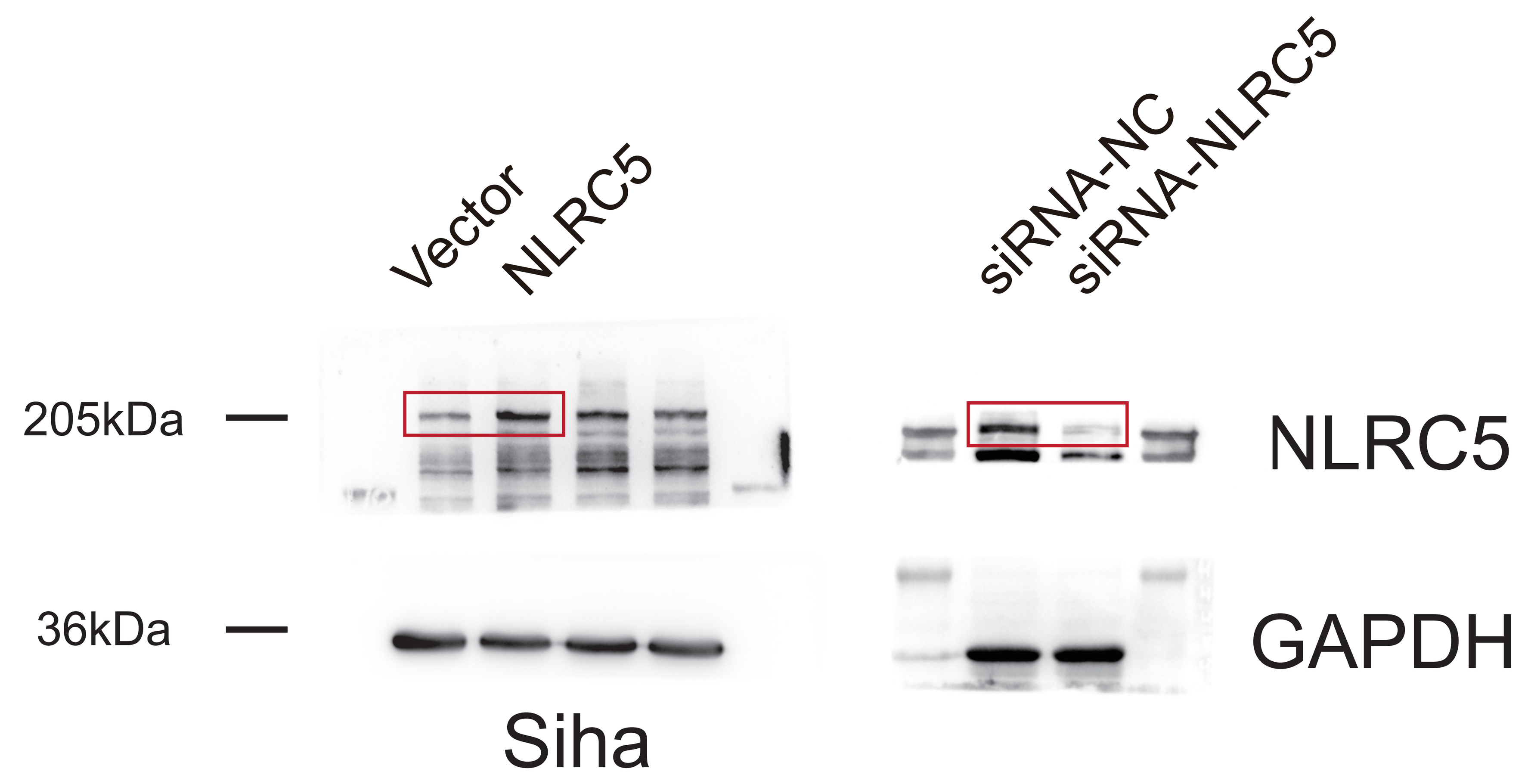


Related to Figure 4a

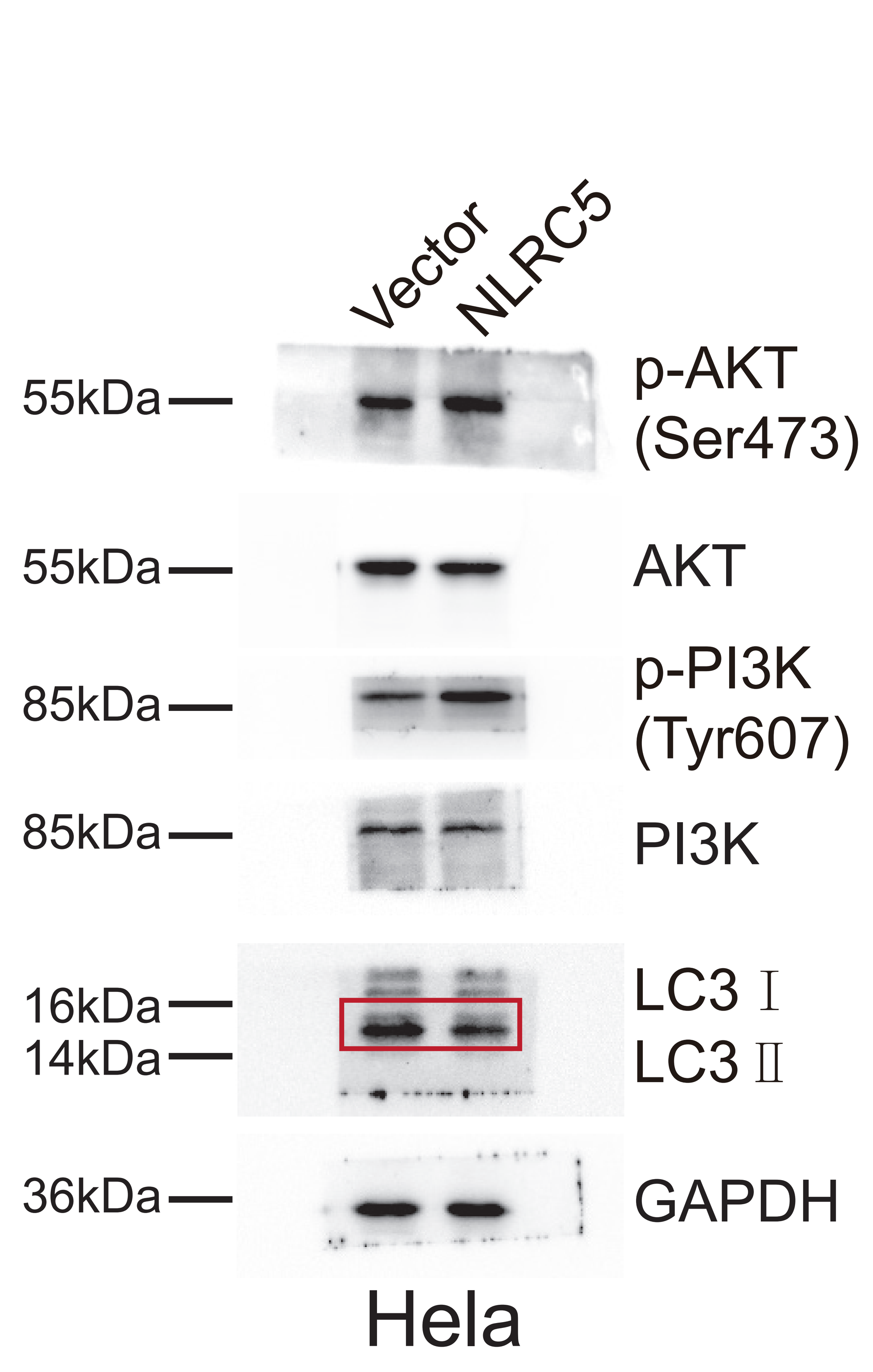


Related to Figure 4b

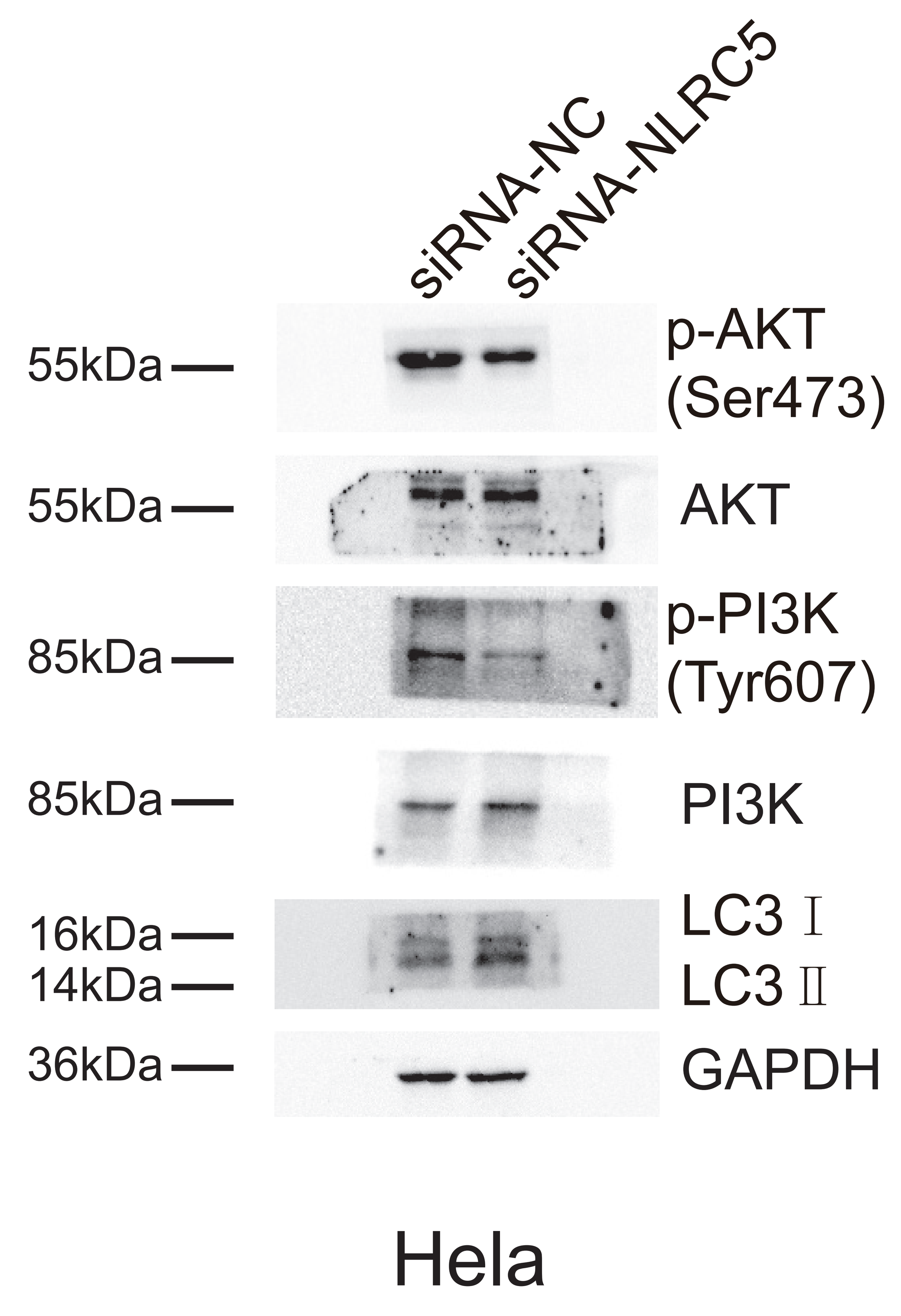


Related to Figure 4c

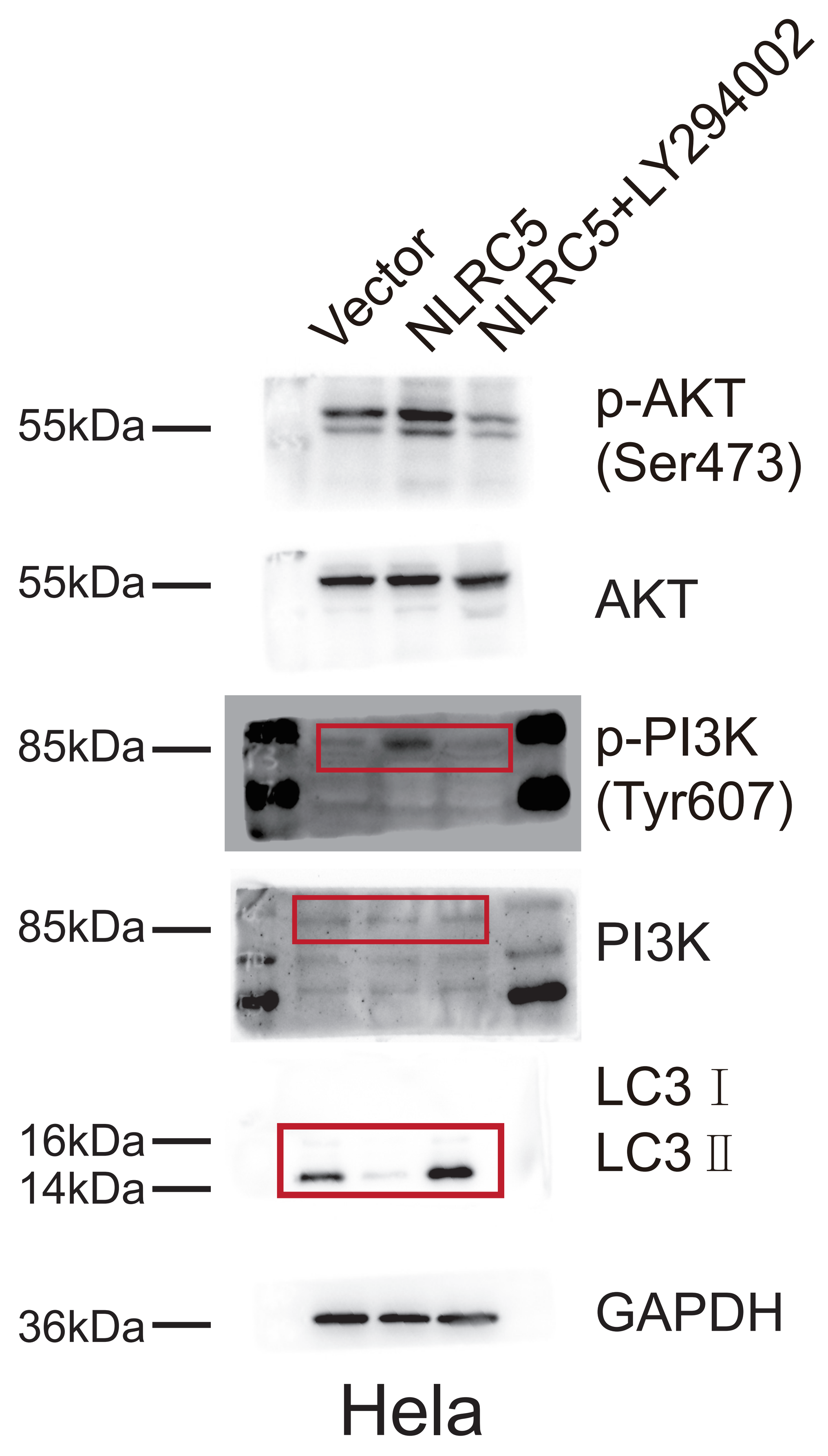


Related to Figure 4e,4f

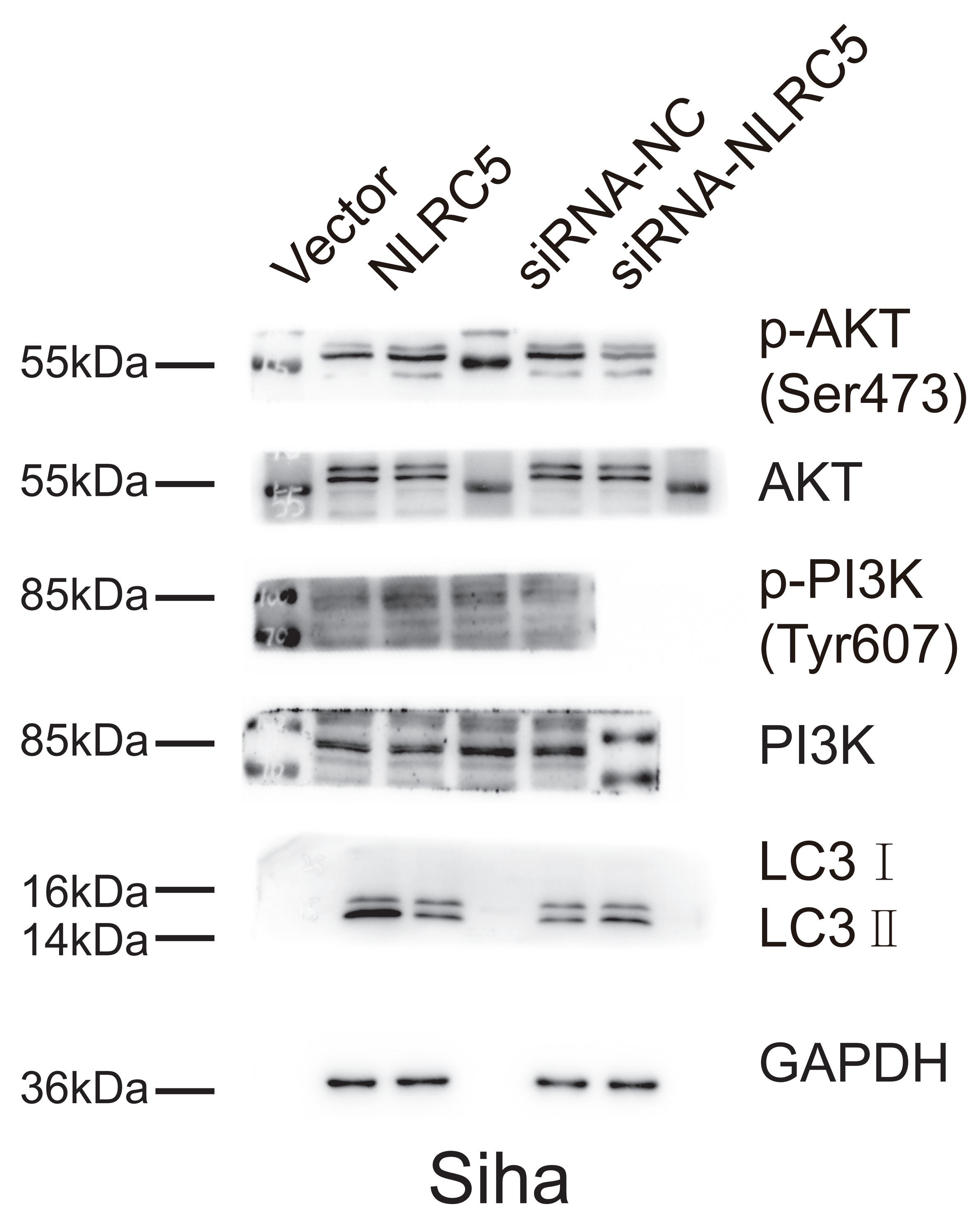


Related to Figure 4g

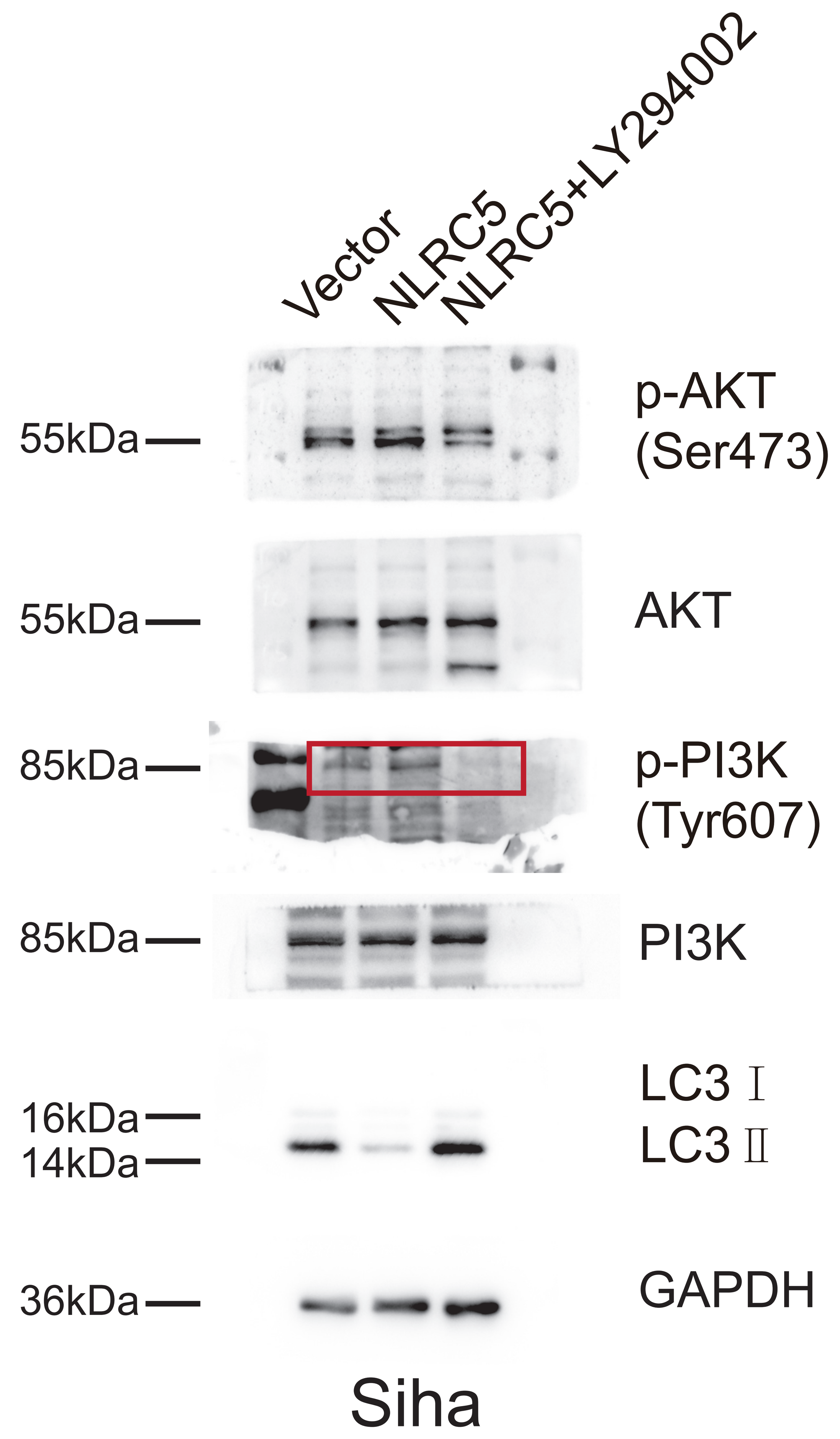

# Original uncropped images of western blots used for the experiment

Repetitions of Western Blots

## Hela

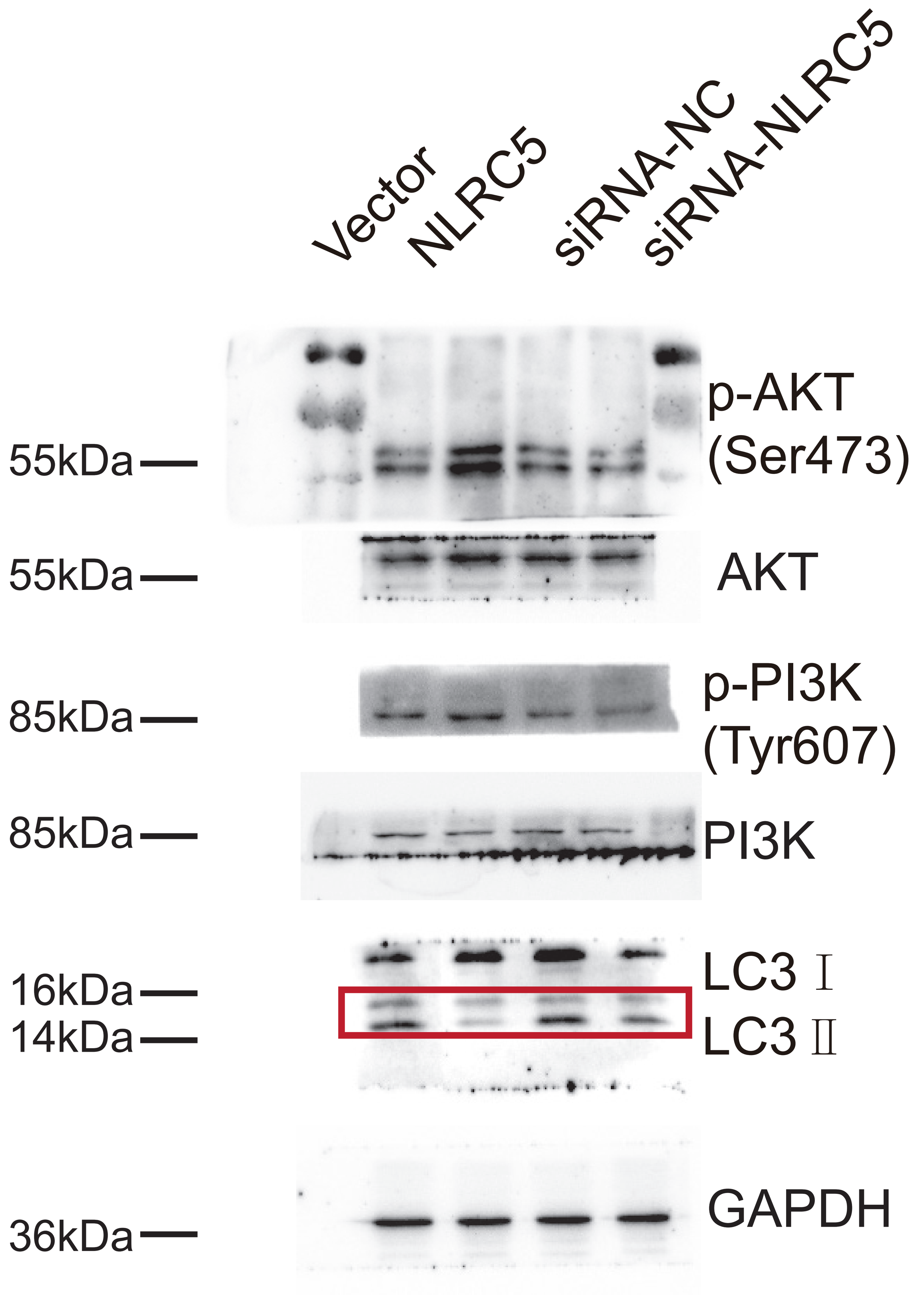


Hela

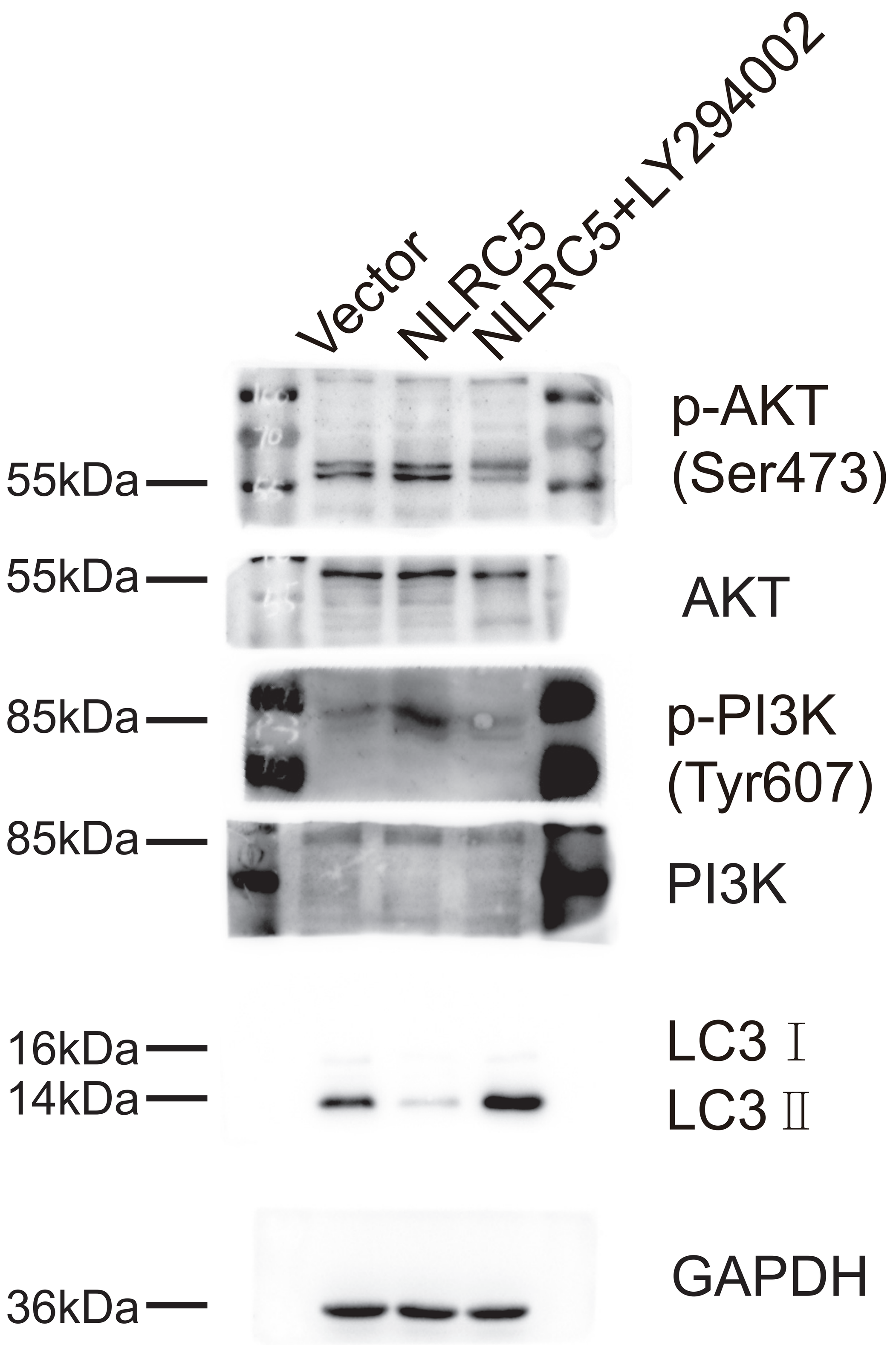


Hela

## Siha

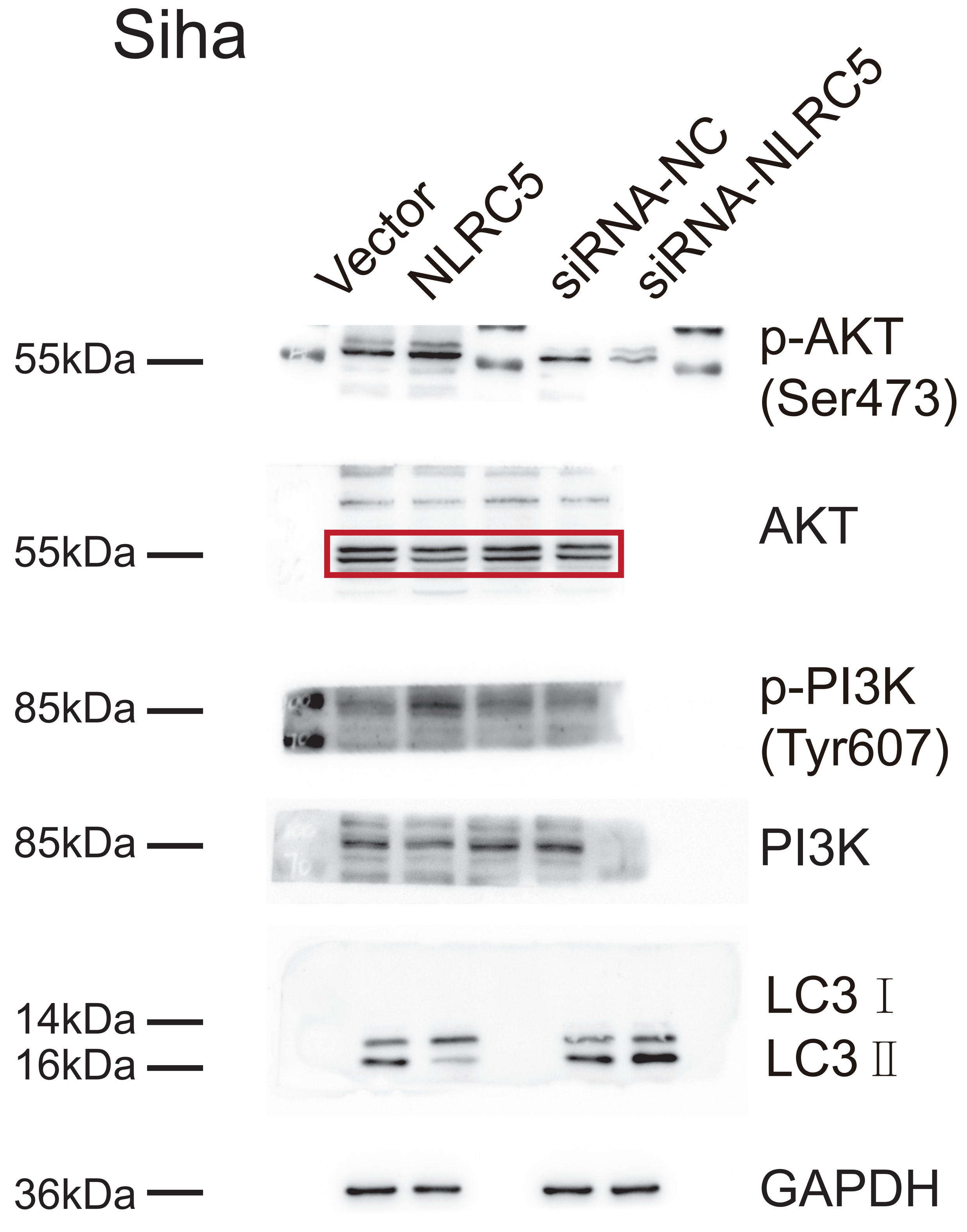


Siha

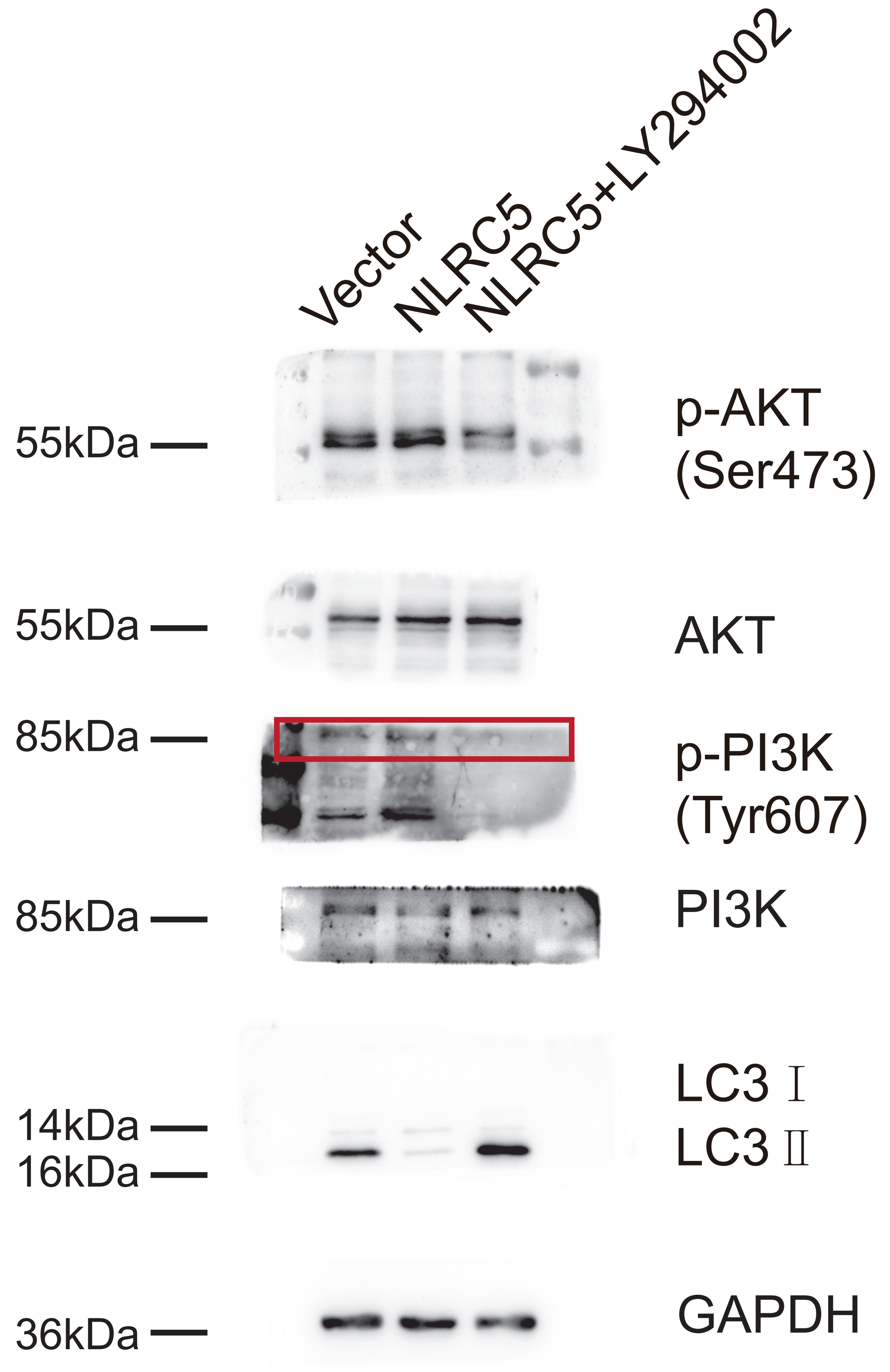


Siha

Original uncropped images of western blots used for the experiment

Additional oligos of siRNAs

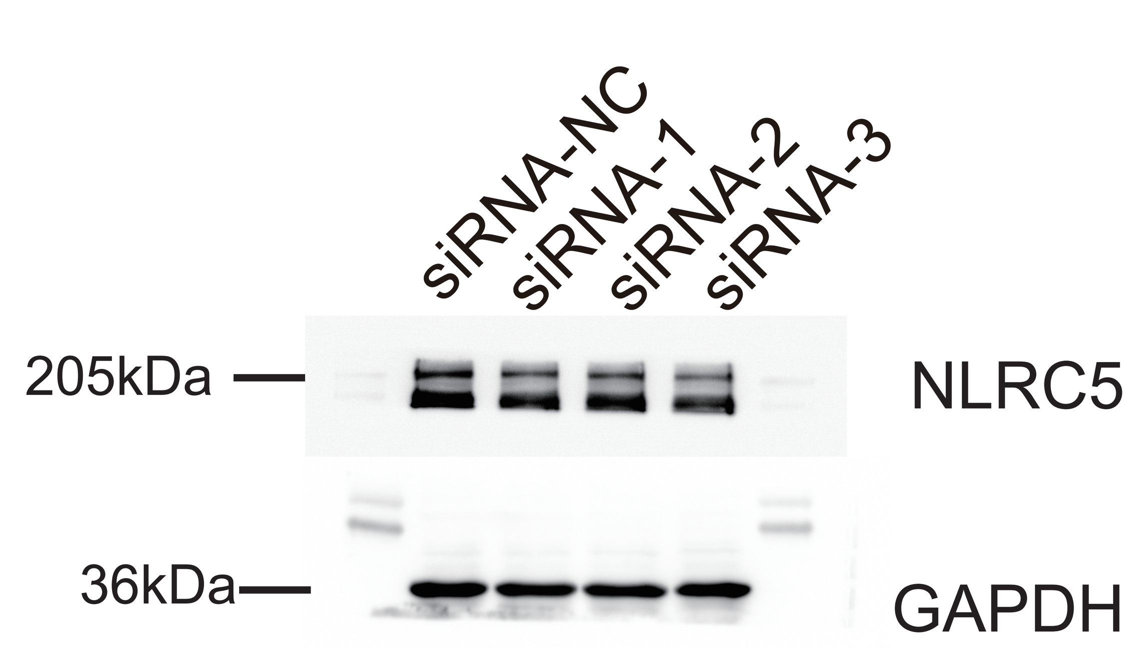


Hela

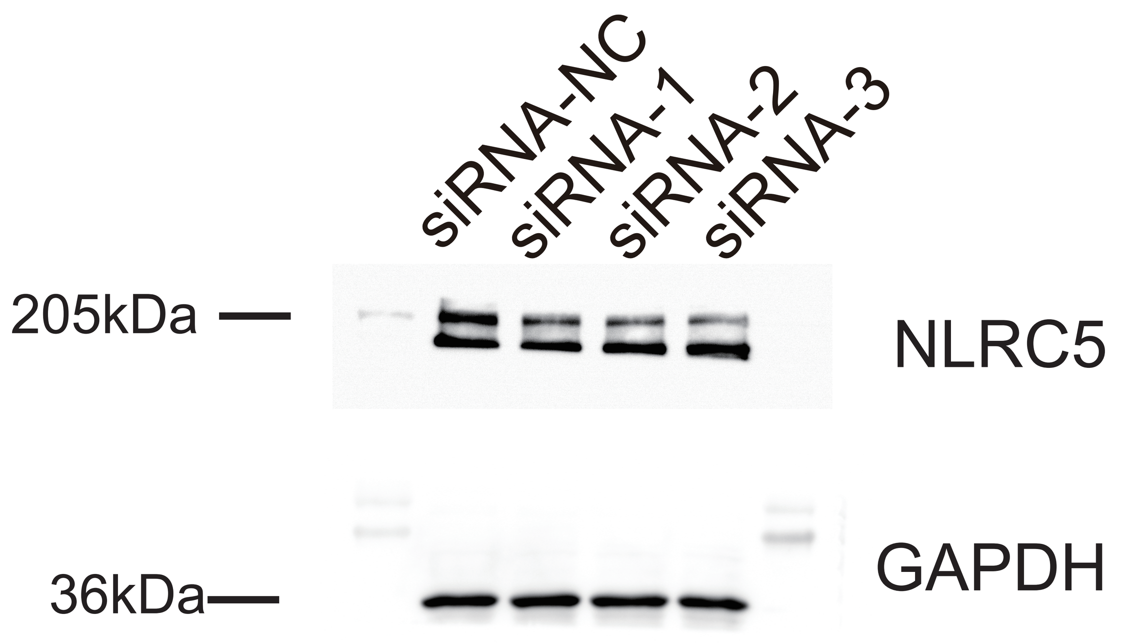


Siha
